# Cellular Activation and Intracellular HCV Load in Peripheral Blood Monocytes Isolated from HCV Monoinfected and HIV-HCV Coinfected Patients

**DOI:** 10.1371/journal.pone.0096907

**Published:** 2014-05-08

**Authors:** Isabelle Dichamp, Wasim Abbas, Amit Kumar, Vincent Di Martino, Georges Herbein

**Affiliations:** 1 Pathogens and Inflammation Department, UPRES EA4266, SFR FED 4234, University of Franche-Comté, Besancon, France; 2 Department of Virology, CHRU Besançon, Besançon, France; 3 Department of Hepatology, CHRU Besançon, Besançon, France; Rush University, United States of America

## Abstract

**Background:**

During HCV infection, the activation status of peripheral blood monocytes and its impact on HCV replication are poorly understood. We hypothesized that a modified activation of peripheral blood monocytes in HIV-HCV coinfected compared to HCV monoinfected patients may contribute to different monocytes reservoirs of HCV replication.

**Methods:**

We performed a case-control analysis involving HCV-infected patients with and without HIV coinfection. In peripheral blood mononuclear cells (PBMCs), peripheral blood lymphocytes (PBLs) and peripheral blood monocytes isolated from HCV monoinfected and HIV-HCV coinfected patients, intracellular HCV load and a marker of cellular activation, nuclear factor-kappaB (NF-κB) activation, were quantified using intracellular detection of HCV-core protein and electrophoretic mobility shift assay, respectively.

**Results:**

Intracellular HCV loads were higher in monocytes isolated from HIV-HCV coinfected patients than in those of monoinfected patients. Among PBMCs isolated from HIV-HCV coinfected patients, intracellular HCV loads were higher in monocytes compared to PBLs. Cellular activation as measured by NF-κB activation was higher in monocytes isolated from HIV-HCV coinfected patients than in those of monoinfected patients.

**Conclusions:**

Our results reveal the peripheral blood monocytes as an important extrahepatic reservoir for HCV in HIV-HCV coinfected patients and indicate a potential association between the activation state of monocytes and the size of the HCV reservoir in HIV-HCV coinfected patients.

## Introduction

Hepatitis C virus (HCV), a positive-strand RNA virus belonging to the Flavivirus, is the major etiologic agent of parenterally-transmitted non-A non-B hepatitis [1]. Currently, almost 3% of the world population is infected by HCV, and these numbers seem to be increasing. One of the most remarkable features of HCV infection is that more than 85% of acutely infected patients become chronically infected. Therefore, in most infected patients, HCV persists indefinitively, leading to chronic hepatitis, cirrhosis, and hepatocellular carcinoma [2]. In addition, HCV is present in approximately one third of patients infected with HIV in developed countries [3]. The accelerated progression of chronic hepatitis C and the increase in life expectancy of HIV-infected patients with the use of combination antiretroviral therapy (HAART) have led to an increase in hospitalizations and deaths attributable to HCV in HIV-HCV-coinfected patients [4]. Several reports found an association between HCV coinfection and progression of HIV disease and HIV infection has also been reported to accelerate the development of severe liver disease [5]–[8].

HCV was originally thought to be a strictly hepatotropic virus, but there is mounting evidence that it can also replicate in peripheral blood mononuclear cells (PBMCs), particularly in patients with HIV infection [9]–[12]. The infected cells were reported to contain HCV negative strand RNA, which is a viral replicative intermediate, and viral genomic sequences were often found to be distinct from those found in serum and liver [13], [14]. Furthermore, it was also reported that several cell types including human T- and B-cell lines, PBMCs, peripheral blood lymphocytes (PBLs) and monocytes/macrophages are capable of supporting HCV infection *in vitro* and *ex vivo* in peripheral blood isolated cells [10], [15]–[18]. In addition, some viral strains were found to be lymphotropic both *in vitro* and *in vivo* in infected chimpanzees [19]. The presence of HCV replication was documented in hematopoietic cells inoculated into the severe combined immunodeficiency mice [20] and in PBMCs from patients after, but not before, liver transplantation [21]. Thus, extrahepatic replication of HCV could be facilitated by immunosuppression.

We report here that in HIV-HCV coinfected patients the peripheral blood monocytes are a main extrahepatic cellular reservoir of HCV and display increased NF-κB activation compared to monocytes isolated from monoinfected patients.

## Patients and Methods

### Patients

We conducted a prospective cohort study of 15 patients, 8 HCV monoinfected patients and 7 HIV-HCV coinfected patients followed in Besancon University Hospital (Table 1). The HCV-infected patients were candidates for pegylated interferon plus ribavirin therapy. They all had history of injecting drug use. The mean age was 59 years (±10 years) and 47 years (±8 years) for monoinfected and coinfected patients, respectively (p = 0.04). The distribution of HCV genotypes, the plasma HCV load and anti-HCV regimen are shown in Table 1. All HIV-positive patients were treated with HAART for at least 1 year, had undetectable plasma HIV-1 RNA levels (<40 copies/ml) for at least 1 year and had a level of CD4+ T lymphocytes higher than 300 cells/mm^3^ of blood. Biological characteristics (CD4+ T cell count, HAART treatment, HIV disease stage) of HIV-infected patients are presented in Table 1. According to the French Regulatory Authority for clinical studies, prospective and retrospective studies with observational analysis only are not evaluated by Human Protection Committees. The Human Protection Committee East Area II from France was consulted and issued a formal waiver of approval. This study did not rely solely on medical records. The authors did not have any contact with the study subjects and performed tests on patient blood samples that were part of a routine care. The blood samples were anonymized before being used by the authors.

**Table 1:** Characteristics of the population studied.

Patient	Age	Gender	HCV Geno type	HIV Status	CD4 count	HAART	Anti-HCV treatment		HIV pVL, copies/ml	Plasma HCV RNA log IU/ml
**HIV-HCV coinfected patients** (n = 7)										
1	39	M	1a	A	1262	D4T,TEN,DDI		0	<40	5.88
2	43	M	4	C	774	3TC,TEN, NEV		0	<40	5.56
3	42	F	3a	B	495	TEN,ABA,FOS,RIT		0	<40	6.30
4	43	F	1a	C	724	LOP, EFA		0	<40	6.15
5	49	M	1a	A	550	AZT,3TC,ABC		0	<40	2.79
6	47	F	1b	A	434	AZT,3TC,ABC		0	<40	6.05
7	65	F	n/a	C	418	TEN, NEV, LOP		0	<40	1.19
Mean	47				665				<40	4.85
**HCV monoinfected patients** (n = 8)										
8	65	F	1a	–	n/a	–	0		0	5.05
9	67	M	1	–	n/a	–	0		0	5.91
10	69	F	3a	–	n/a	–	0		0	5.78
11	52	F	1	–	n/a	–	0		0	5.55
12	42	F	n/a	–	n/a	–	0		0	2.79
13	56	M	n/a	–	n/a	–	0		0	6.33
14	49	F	1b	–	n/a	–	0		0	5.88
15	74	M	1	–	n/a	–	0		0	6.31
Mean	59									5.45

n/a, not available.

### Isolation and Culture of PBMCs, PBLs and Monocytes

Isolation of PBMCs was done by Ficoll gradient centrifugation, as previously reported [22]. Peripheral blood from patients was diluted with equal amounts of PBS, was overlaid on Ficoll medium (Eurobio, Les Ulis, France), and was centrifuged at 900×g for 30 min at 25°C without break and acceleration. The PBMC band was removed and washed 2 times with PBS. Cell count was determined by Malassez cytometer (Poly Labo, Strasbourg, France) and resuspended in RPMI-1640 medium without addition of serum. The cells were plated onto plastic cell culture flasks and incubated at 37°C. After 2 h, the nonadherent cells were removed to get peripheral blood lymphocyte (PBL)-enriched culture. Adherent cells (>95% CD14^+^ by flow cytometric analysis), monocytes, were washed with sterile PBS and cultured in RPMI-1640 medium supplemented with 10% (v/v) human AB serum, penicillin (100 IU/ml), and streptomycin (100 µg/ml).

### Electrophoretic Mobility Shift Assay (EMSA)

To measure the NF-κB activation, EMSA was carried out as previously described [23]. Briefly, nuclear extracts prepared from PBMCs, PBLs and monocytes were incubated with 20 fmol of biotin-end-labeled 45bp NF-κB oligonucleotide, 5-TTGTTACAA**GGGACTTTC**CGCTG**GGGACTTTC**CAGGGAGGCGTGG-3 (bold indicates NF-κB binding sites) in the presence of binding buffer [10 mMTris, 50 mMKCl, 1 mM DTT at pH 7.5 and 50 ng/µlPoly (dI•dC)]. NF-κB oligonucleotide was labeled with biotin using Biotin 3′ End DNA Labeling kit (Pierce, Rockford, IL) and complementary pairs were annealed by heating in boiling water for 5 min and then reducing the temperature slowly till room temperature. The DNA-protein complex formed was resolved from free oligonucleotide on a 6% native polyacrylamide gel in 1X Tris-borate-EDTA buffer, using Mini-PROTEAN 3 Cell (Bio-Rad, Hercules, CA) and was transferred to Biodyne precut nylon membrane (Pierce) using Mini Trans-Blot Electrophoretic Transfer Cell (Bio-Rad). Biotin-end-labeled DNA was detected by LightShift Chemiluminescent EMSA kit (Pierce).

### Serological and Virological Markers

HIV infection was assessed by the positivity of two serological tests including ELISA (HIV Genscreen ULTRA, Biorad; HIV Duo Roche, Basel, Switzerland) and Western blot (HIV Blot 2.2, MP Diagnostics, Solon, OH). Quantification of plasmatic HIV RNA was done using a COBAS TaqMan HIV-1 assay (Roche). HCV infection was assessed by the positivity of two serological tests (Monolisa HCV Ag-Ab ULTRA Bio-Rad, Roche anti-HCV assay). The quantification of plasmatic HCV RNA was done by a bDNA assay (Quantiplex HCV Versant 3.0, Bayer, Leverkusen, Germany). The intracellular detection of capsid antigen and antibodies associated with an infection by HCV was done with the Monolisa HCV Ag-Ab ULTRA assay that is an immunoassay for the detection of HCV infection (Biorad) [24].

### Statistical Analysis

Figures show the means of independent experiments and standard deviations. Statistical analysis was performed using the Mann Whitney U test and considered significant at p≤0.05. The program used for plotting was Microsoft Excel.

## Results

### Comparison of Intracellular HCV Load in PBMCs Isolated from HIV-HCV Coinfected Patients and Mono-infected Patients

**Figure 1:**
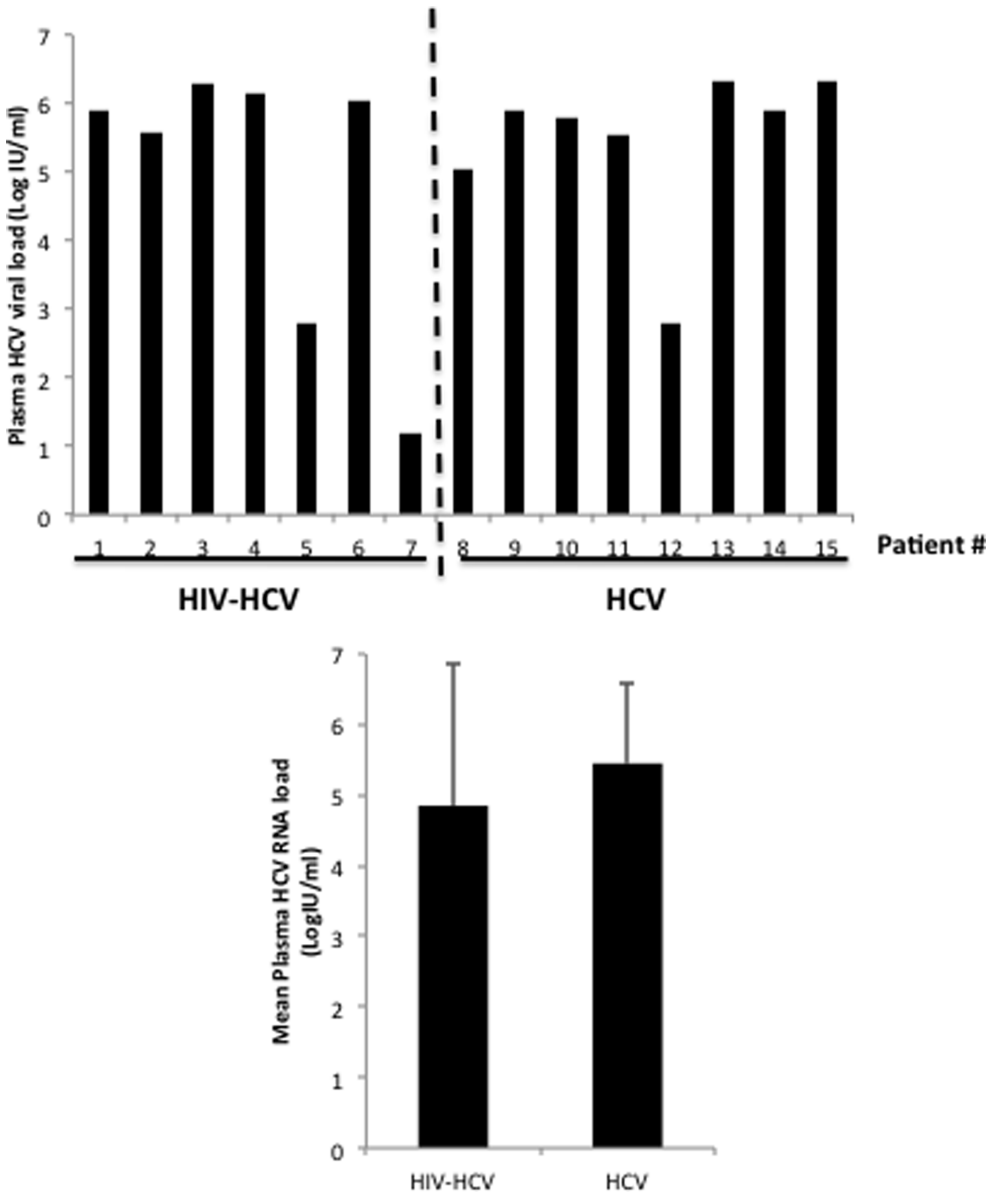
Plasma HCV loads in HIV-HCV coinfected patients and in HCV monoinfected subjects. Individual values (upper panel) and mean values (±S.D.) (lower panel) of plasma HCV RNA loads were measured. p = NS.

**Figure 2:**
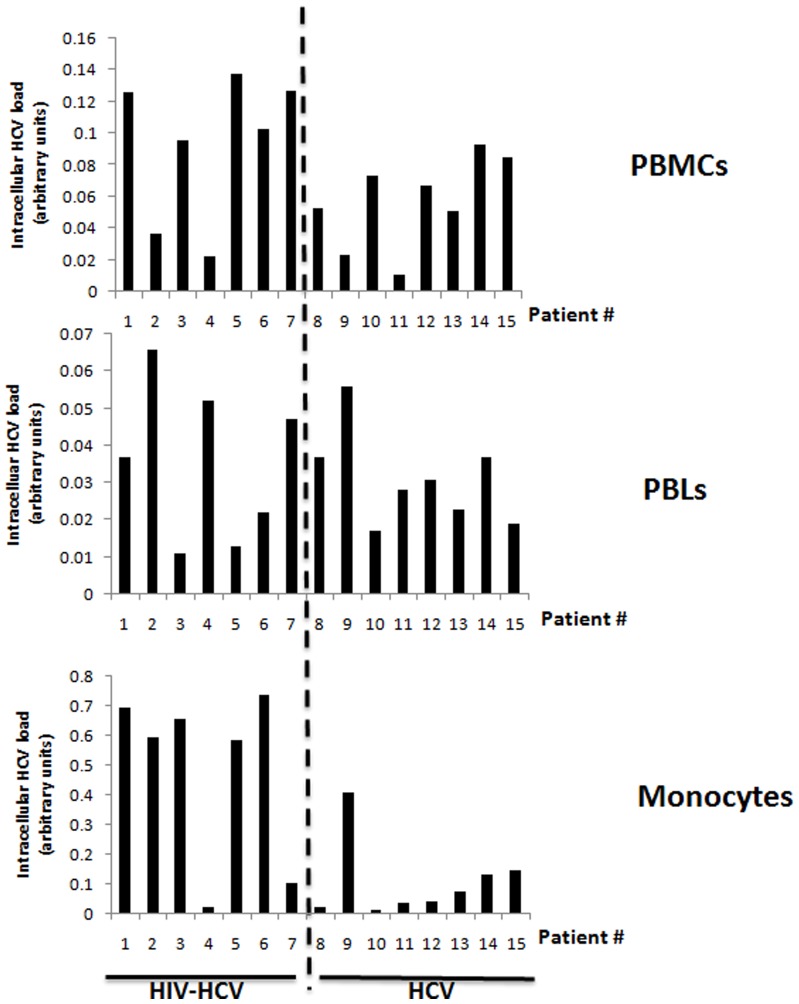
Intracellular HCV loads in HIV-HCV coinfected patients and in HCV monoinfected subjects. Intracellular HCV loads were measured in autologous PBMCs, PBLs and monocytes isolated from the peripheral blood of monoinfected and coinfected patients as described in Materials and Methods. Please note the different scales of *y* axis used for each cell population.

**Figure 3:**
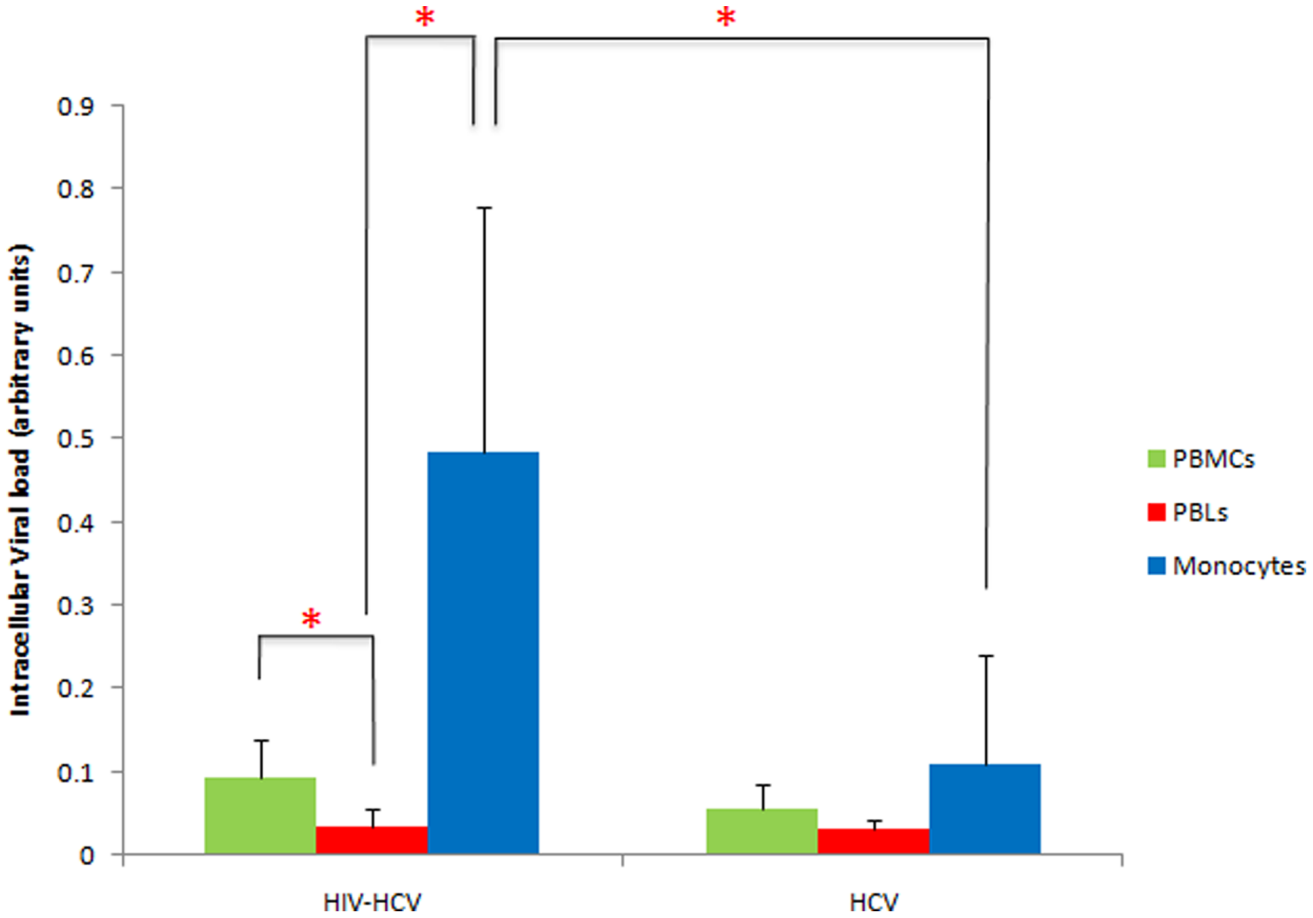
Preferential detection of intracellular HCV in peripheral blood monocytes of HIV-HCV coinfected patients. Means (±S.D.) of intracellular HCV loads measured in autologous PBMCs, PBLs and monocytes isolated from the peripheral blood of HCV monoinfected and HIV-HCV coinfected patients as described in Materials and Methods are indicated. *p≤0.05.

We measured both the plasma and intracellular HCV load in PBMCs isolated from HIV-HCV coinfected patients and HCV monoinfected patients. The mean plasma HCV load was 5.45 log IU/ml (±1.07) in HCV monoinfected patients and 4.85 log IU/ml (±1.86) in HIV-HCV coinfected patients (p = NS) (Figure 1, Table 1). The mean intracellular HCV load in PBMCs was not significantly different among HIV-HCV coinfected patients and monoinfected patients (0.093 OD vs. 0.057 OD, p = 0.09) (Figures 2 and 3).

### Comparison of Intracellular HCV Load in Monocytes and PBLs from HIV-HCV Coinfected and HCV Monoinfected Patients

To determine the subset(s) of mononuclear cells that harbor HCV, we separated monocytes from autologous PBLs isolated from the peripheral blood of HIV-HCV coinfected patients and from HCV infected patients. We measured in both cell types the intracellular HCV viral load (Figures 2 and 3). In coinfected patients, the intracellular HCV load was 13.8-fold higher in monocytes than in PBLs (0.485 OD vs. 0.035 OD, p = 0.01) (Figure 3). In HCV monoinfected subjects, the intracellular HCV load was not significantly different in monocytes and in PBLs (0.111 OD vs. 0.031 OD, p = 0.12) (Figure 3). Thus, our results indicate that monocytes rather than PBLs harbor HCV in coinfected patients (Figure 2 and 3). Moreover, the intracellular HCV load was 4.4-fold higher in monocytes of coinfected subjects than in monocytes of monoinfected subjects (0.485 OD vs. 0.111 OD, p = 0.037) (Figure 3).

### Higher NF-κB Activation in Monocytes from HIV-HCV Coinfected Patients Compared to Monocytes from Monoinfected Patients

**Figure 4:**
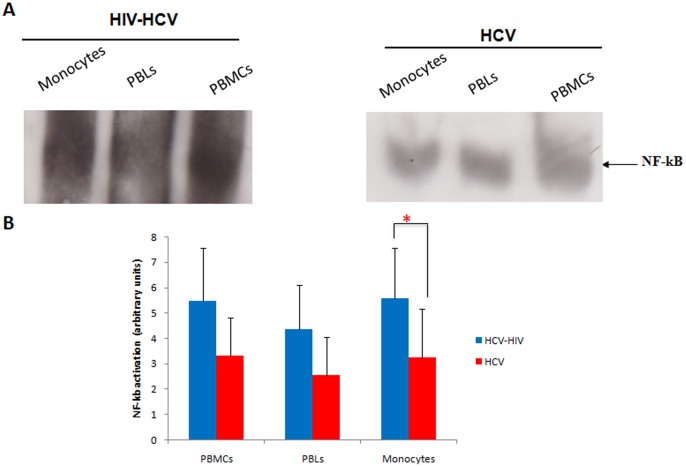
Measurement of NF-κB activation in PBMCs, PBLs and monocytes isolated from the peripheral blood of HIV-HCV coinfected patients and HCV monoinfected patients. (A) NF-κB activation measured by EMSA in PBMCs, PBLs and monocytes isolated from the peripheral blood of a coinfected patient and a monoinfected patient. Results are representative of twelve independent experiments (7 coinfected patients, 5 monoinfected patients). (B) Mean values (± S.D.) of NF-κB activation in PBMCs, PBLs and peripheral blood monocytes isolated from coinfected patients and monoinfected patients, using an EMSA followed by quantification with a phosphoimager as described in Materials and Methods. *p≤0.05.

The activation state of monocytes can be assessed by the expression of cell surface markers such as up-regulation of CD69 and HLA-DR and the release of soluble CD14, but also by the specific activation of intracellular pathways such as NF-κB activation [25], [26], [27]. Since HCV replication is inhibited by interferon that could be regulated through NF-κB-dependent mechanisms [28]–[30] and since HIV activates NF-κB in several cell types including monocytes/macrophages [7], [31], [32], we assessed the level of NF-κB activation in monocytes, but also in autologous PBLs and PBMCs isolated from the peripheral blood of coinfected patients and HCV monoinfected patients. We measured NF-κB activation using an EMSA followed by quantification with a phosphoimager as previously reported [23] (Figure 4A). Although levels of NF-κB activation were not statistically different in PBMCs of coinfected and monoinfected patients (5.47 versus 3.30, p = 0.10), a 1.7-fold higher NF-κB activation was measured in monocytes of HIV-HCV coinfected subjects compared to monocytes isolated from HCV monoinfected patients (5.60 versus 3.25, p = 0.04) (Figure 4B). In contrast to monocytes, levels of NF-κB activation were not statistically different in PBLs of coinfected and monoinfected patients (4.36 versus 2.54, p = 0.07) (Figure 4B). Our results indicate high intracellular HCV loads and high levels of NF-κB activation in monocytes isolated from HIV-HCV coinfected patients.

## Discussion

We observed higher intracellular HCV loads in monocytes isolated from HIV-HCV coinfected patients compared to monocytes isolated from monoinfected patients. Higher NF-κB activation was measured in monocytes of HIV-HCV coinfected patients compared to monocytes isolated from HCV monoinfected patients. Our results underline the peripheral blood monocytes as an important extrahepatic reservoir for HCV in HIV-HCV coinfected patients and suggest that monocytes activation could participate to the formation of the HCV reservoir.

In our study, we observed similar levels of plasma HCV load in monoinfected and coinfected patients. This result was not surprising given the normal levels of CD4 cell count in our HIV-infected population [6]. However besides plasma viral load, the detection of HCV infection in cellular subpopulations of the peripheral blood was important [33] and able to discriminate substantial differences between HCV monoinfected and HIV-HCV coinfected patients. Whereas there is little doubt that HCV replicates primarily in the liver, the presence of extrahepatic replication sites remains controversial. This evidence has been questioned because commonly used techniques are limited in their ability to discriminate between positive and negative strands. In several earlier studies that used assays optimized for strand specificity, HCV negative strand RNA was not detected in PBMCs from infected patients [34], [35]. By contrast others have recently reported the relatively common detection of HCV negative strand RNA in PBMCs [10], [15], [18]. We used a HCV core antigen ELISA assay, since it was reported that monitoring of viral kinetics by use of either core antigen or RNA concentrations in HCV-infected patients undergoing antiviral combination therapy resulted in very similarly shaped curves in all cases [36]. Additionally, the HCV core antigen ELISA detected intracellular virus and not cell-bound virus, since similar levels of HCV core antigen were detected in monocytes from HCV-infected patients with or without trypsin treatment (data not shown). We detected only very low amounts of HCV in PBMCs of monoinfected and coinfected patients. Therefore, we decided to assess the presence of HCV in PBMC subpopulations, namely peripheral blood monocytes and PBLs. We observed primarily the presence of HCV in the peripheral blood monocytes, and almost not in PBLs, isolated from HIV-HCV coinfected patients. In agreement with our data, within the population of PBMCs, among the cells harboring replicating HCV, monocytes/macrophages have been reported previously to be potentially one of the main cellular targets [10], [13]. Although the HCV infection of monocytes was constantly observed in both monoinfected and coinfected patients, we measured the highest amounts of HCV in monocytes of coinfected patients. This might indicate that HIV infection favors the replication of HCV in monocytes of coinfected patients [9], even in HAART-treated patients with undetectable plasma HIV load.

Beside the preferential distribution of HCV in monocytes of coinfected patients, we observed higher levels of NF-κB activation in monocytes of coinfected patients compared to monocytes of monoinfected patients. NF-κB activation is increased in HIV-infected T-cells and monocytes/macrophages and favors HIV-1 replication [32]. The HIV-1 proteins, such as Nef, Vpr, and Tat stimulate NF-κB activation through the RelA/p50 canonical pathway in monocytes/macrophages and T-cells, respectively [23], [31], [37]. We and other teams recently reported that the HIV-1 Nef and HCV Core proteins stimulate additionally NF-κB activation and favor both HIV-1 replication in monocytes/macrophages and hepatic fibrogenesis [7], [38], [39]. Our results indicate that the high levels of NF-κB activation observed in monocytes of coinfected patients are concomitant of high intracellular HCV loads. We also observed the highest levels of the activation marker HLA-DR on monocytes isolated from HCV-infected patients as compared to healthy subjects (data not shown), indicating the potential use of HLA-DR marker as an activation marker on peripheral blood cells of HCV-infected and/or coinfected patients [26], [27]. Additionally, markers of innate immune activation such as soluble CD14 predict poor host response to interferon-alpha-based HCV therapy during HIV-HCV coinfection [40], [41]. Although intracellular HCV load is enhanced in monocytes of HIV-HCV coinfected patients, it is unclear whether HIV facilitates HCV infection directly or indirectly as a consequence of immunosuppression. Our study is a proof-of-concept study on a limited number of patients. Future clinical trials will be designed to unveil the molecular mechanism(s) involved in HCV replication in monocytes/macrophages.

In absence of coinfection, HCV infection usually down-regulates NF-κB activation directly via viral proteins such as HCV Core or indirectly through inactivation of the MAVS (mitochondrial antiviral signaling) protein [42]–[44]. We observed that monocytes isolated from HCV monoinfected patients display lower levels of NF-κB activation compared to monocytes isolated from HIV-HCV coinfected patients. Several studies confirm that in the absence of HIV infection, the optimal replication of HCV requires low levels of NF-κB activation. Sustained NF-κB activation has been reported to be a major factor for the impediment of HCV replication [29]. HCV triggers activation of the dsRNA-dependent eIF2a kinase PKR which leads to the inhibition of IFN expression through general control of translation while the viral genome can be translated from its eIF2a-insensitive IRES structure [28], [30]. Interestingly PKR silencing suppresses NF-κB activation in Huh7.5.1 cells, indicating that the modulation of HCV replication by PKR is dependent on NF-κB mediated interferon response [29].

Since enhanced NF-κB activation favors the production of proinflammatory cytokines and chemokines in monocytes/macrophages and results in enhanced cellular activation [45], the low-levels of NF-κB activation observed in HCV-harboring monocytes isolated from monoinfected patients will rather lead to a state of cellular deactivation [46]. Other defects in innate immunity have been reported in HCV infection [11], [47], [48]. HCV structural proteins can interact with TLR-2 in monocytes and induce IL-10 production, which blocks NF-κB activation in monocytes by an autocrine feedback loop and which inhibits IFN-alpha and IL-12 production in dendritic cells by a paracrine mechanism [48]. *In vitro*, TLR2 and TLR4 activation by the HCV core protein leads to a decrease in interleukin-6 production by human antigen-presenting cells by the negative regulation of NF-κB activation by the induction of IRAK-M [42]. Additionally, TLR ligand-induced IL-6 production is significantly reduced in peripheral blood monocytes isolated from HCV-infected patients, compared with those of healthy control subjects [42]. Therefore, optimal HCV replication in monoinfected patients could require deactivated monocytes that might be part of a more general failure of innate immunity in these patients [9], [11]. The use of anti-HCV proteases could modify the size of the cellular reservoir in extrahepatic sites and will require future studies [49]–[51].

Our results reveal the peripheral blood monocytes as a potential important extrahepatic HCV reservoir in HIV-HCV coinfected patients and suggest that monocyte activation could participate to the formation of the HCV reservoir in HIV-HCV coinfected patients. This might have important therapeutic implications for the clearance of HCV from cellular reservoirs in HIV-HCV coinfected patients.
